# Subchondral defects resembling osteochondrosis dissecans in joint surfaces of the extinct saber-toothed cat *Smilodon fatalis* and dire wolf *Aenocyon dirus*

**DOI:** 10.1371/journal.pone.0287656

**Published:** 2023-07-12

**Authors:** Hugo Schmökel, Aisling Farrell, Mairin F. Balisi

**Affiliations:** 1 Evidensia Academy, Division of Orthopaedics, Stockholm, Sweden; 2 La Brea Tar Pits and Museum, National History Museums of Los Angeles County, Los Angeles, California, United States of America; 3 Raymond M. Alf Museum of Palaeontology, Claremont, California, United States of America; University of Silesia, POLAND

## Abstract

Skeletal disease may hamper the behavior of large predators both living and extinct. We investigated the prevalence of osteochondrosis dissecans (OCD), a developmental bone disease affecting the joints, in two Ice Age predators: the saber-toothed cat *Smilodon fatalis* and dire wolf *Aenocyon dirus*. As published cases in modern Felidae and wild Canidae are rare, we predicted that subchondral defects resembling OCD would be rare in the extinct predators. We examined limb joints in juvenile and adult *S*. *fatalis*: 88 proximal humeri (shoulder), 834 distal femora (stifle), and 214 proximal tibiae. We also examined limb joints in juvenile and adult *A*. *dirus*: 242 proximal humeri, 266 distal femora, and 170 proximal tibiae. All specimens are from the Late Pleistocene Rancho La Brea fossil locality in Los Angeles, California, USA. While the *Smilodon* shoulder and tibia showed no subchondral defects, subchondral defects in the *Smilodon* femur had a prevalence of 6%; most defects were small (<7mm); and nine adult stifles with defects also showed osteoarthritis. Subchondral defects in the *A*. *dirus* femur had a prevalence of 2.6%; most defects were large (>12mm); and five stifles further developed mild osteoarthritis. Subchondral defects in the *A*. *dirus* shoulder had a prevalence of 4.5%; most defects were small, and three shoulders developed moderate osteoarthritis. No defects were found in the *A*. *dirus* tibia. Contrary to our prediction, we found a high prevalence of subchondral defects in the stifle and shoulder of *S*. *fatalis and A*. *dirus* resembling OCD found in humans and other mammals. As modern dogs affected by OCD are highly inbred, this high prevalence in the fossil taxa may suggest that they experienced inbreeding as they approached extinction. The deep-time history of this disease supports the need for monitoring of animal domestication, as well as conservation, to avoid unexpected surges in OCD under conditions like inbreeding.

## Introduction

Osteochondrosis (OC) is an orthopedic disease caused by a failure of the endochondral ossification in the epiphyseal plate and joint cartilage in a variety of species ranging from horses to humans. It is associated with the complex of developmental orthopedic pathologies, causing lameness in advanced cases [1–4]. One manifestation of OC is osteochondrosis dissecans (OCD), which is considered in the failure of cellular differentiation in growing cartilage, leading to its thickening or retention. Trabecular damages around the thickened cartilage appear and will develop into a sclerotic ring visible by radiology. The cartilage flap can detach into the joint cavity, and the detached fragments and the exposure of subchondral bone will induce joint inflammation, which can lead to subsequent painful secondary osteoarthritis (OA) and hinder joint motion [3, 4].

While developmental skeletal diseases like osteochondrosis are well documented as markers of health in domestic animals [4], fewer studies have examined the extent to which they affect wild animals, even in captivity. Radiographic examination of living wild animals is costly; and, to our knowledge, only a few institutions house wildlife skeletal postcranial collections large enough to permit reconstructing the prevalence of osteochondrosis or other developmental skeletal disorders in a population. However, developmental skeletal diseases have been suggested to impact the extinct Ice Age saber-tooth cat *Smilodon fatalis* [5, 6], at least 2,000 individuals of which are preserved together with at least 4,000 individuals of the dire wolf *Aenocyon dirus* (formerly *Canis dirus*) at the Rancho La Brea (RLB) asphalt seeps in Los Angeles, California, USA. While a variety of taphonomic scenarios as well as asphaltic movement over tens of thousands of years have preserved mostly disarticulated skeletons at RLB, the uncommonly large sample sizes enable documentation of the prevalence and distribution of bone and joint diseases—including, possibly, osteochondrosis—in these fossil species [7].

The Rancho La Brea asphalt seeps primarily functioned as a carnivore trap: a large herbivore mired in the asphalt inadvertently would attract large carnivores and scavengers, which themselves would become entrapped in great numbers. Studies of *Smilodon fatalis* and *Aenocyon dirus* at RLB have reconstructed their dietary specialization on herbivorous megafauna, with *Smilodon* probably being primarily an ambush predator and *Aenocyon* a pursuit predator [8, 9]. The abundant specimens at RLB include numerous examples of bone lesions, or pathologies, incurred by trauma to the animals likely as they hunted large prey; the distribution of these traumatic injuries on the skeleton support the locomotor inferences based on comparative morphology [7].

These specimens were excavated over the last century from more than 100 deposits or “pits” that, together, present a picture of the several thousand years leading up to the Late Pleistocene extinctions between 12,000 and 10,000 years ago, when *Smilodon*, *Aenocyon*, and other Ice Age megafauna disappeared from North America [10–13].

OCD is a commonly found orthopedic disease in modern dogs, mainly affecting the shoulder joint as well as the stifle and the tibio-tarsal joint [14]; but published evidence of the disease is largely absent in wild dogs. Meanwhile, only a few publications have documented OC/OCD in domestic and wild cats, suggesting that this condition is rare in felids. In these published cases, OCD clinically affected the cats, and the defect was in the lateral femoral condyle of the large Felid *Panthera uncia* [15–17]. Intact femora and humeri including the epiphysis of *S*. *fatalis* and *A*. *dirus* are well preserved in the RLB collections, making it possible to examine the joint surfaces for subchondral defects and secondary osteoarthritic pathologies like osteophyte formation, eburnation, and periosteal reactions. Given the rarity of published cases in modern Felidae and wild Canidae, we predicted that subchondral defects resembling OCD also would be rare in these extinct Ice Age predators.

## Material and methods

All specimens were excavated at the Rancho La Brea site and processed at the laboratory in the museum. They are housed at the La Brea Tar Pits Museum. The La Brea Tar Pits Museum is a sister institution of the Los Angeles County Museum (LACM). No permits were required for the described study, which complied with all relevant regulations.

Juvenile distal femoral epiphyses and the distal femoral joint surfaces of adult *S*. *fatalis* and *A*. *dirus* found in Pits 3, 4, and 61/67 at RLB were inspected (S1 Table). Additionally, the proximal humeral joint surfaces of adult and juvenile *S*. *fatalis and A*. *dirus* from the same RLB collection were inspected (S2 Table). The proximal joint surfaces of *S*. *fatalis* and *A*. *dirus* tibiae from Pits 3, 60, and 61 were also examined. Pathologies not related to a subchondral defect were recorded (S3 and S4 Tables). Specimens with joint surfaces that were damaged postmortem were excluded from the study group. Subchondral defects were documented as small (<7mm), medium (7-12mm), and large (>12mm). Joint pathologies were recorded, and osteoarthritic (OA) changes were documented in adult specimens analog to radiographic OA scoring as mild, moderate, and severe [18]. The correlation between defect size and OA score, both ordinal variables, was calculated with a Spearman’s test. Radiographs of epiphyseal specimens were taken at the Natural History Museum of Los Angeles County, Los Angeles, California, USA, using a Faxitron Cabinet X-ray System (Model 43855C). All statistical analysis and visualization were conducted in R 4.2.2 (R Core Team, 2022).

## Results

### Smilodon fatalis

A total of 413 intact adult distal femora and 421 juvenile femoral epiphyses, 214 proximal tibiae and 88 proximal humeral joints from *S*. *fatalis* were included in the study group.

**Fig 1:**
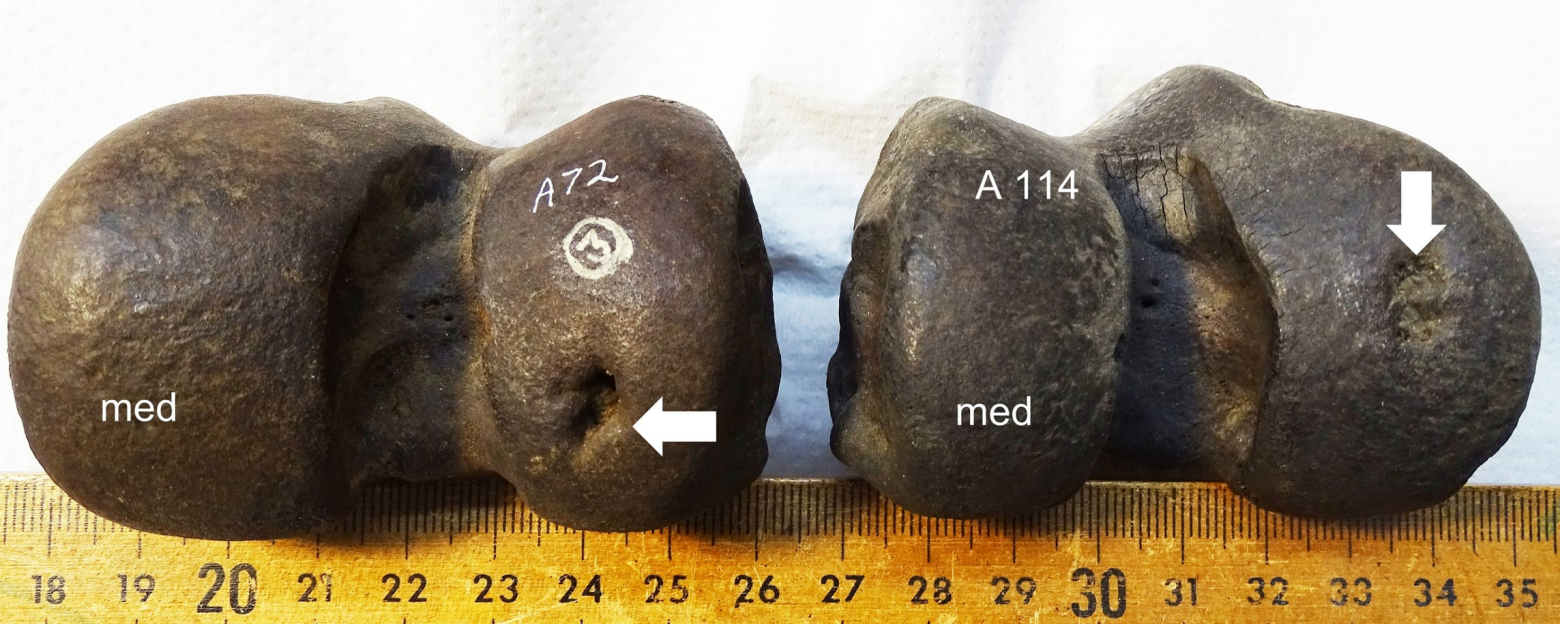
Subchondral defects in juvenile *S*. *fatalis* femoral joint surfaces of specimen LACMHC A72 and LACMHC A114, medium defect size, location lateral center, no secondary osteoarthritis. Notice the difference in depth of the defect.

**Fig 2:**
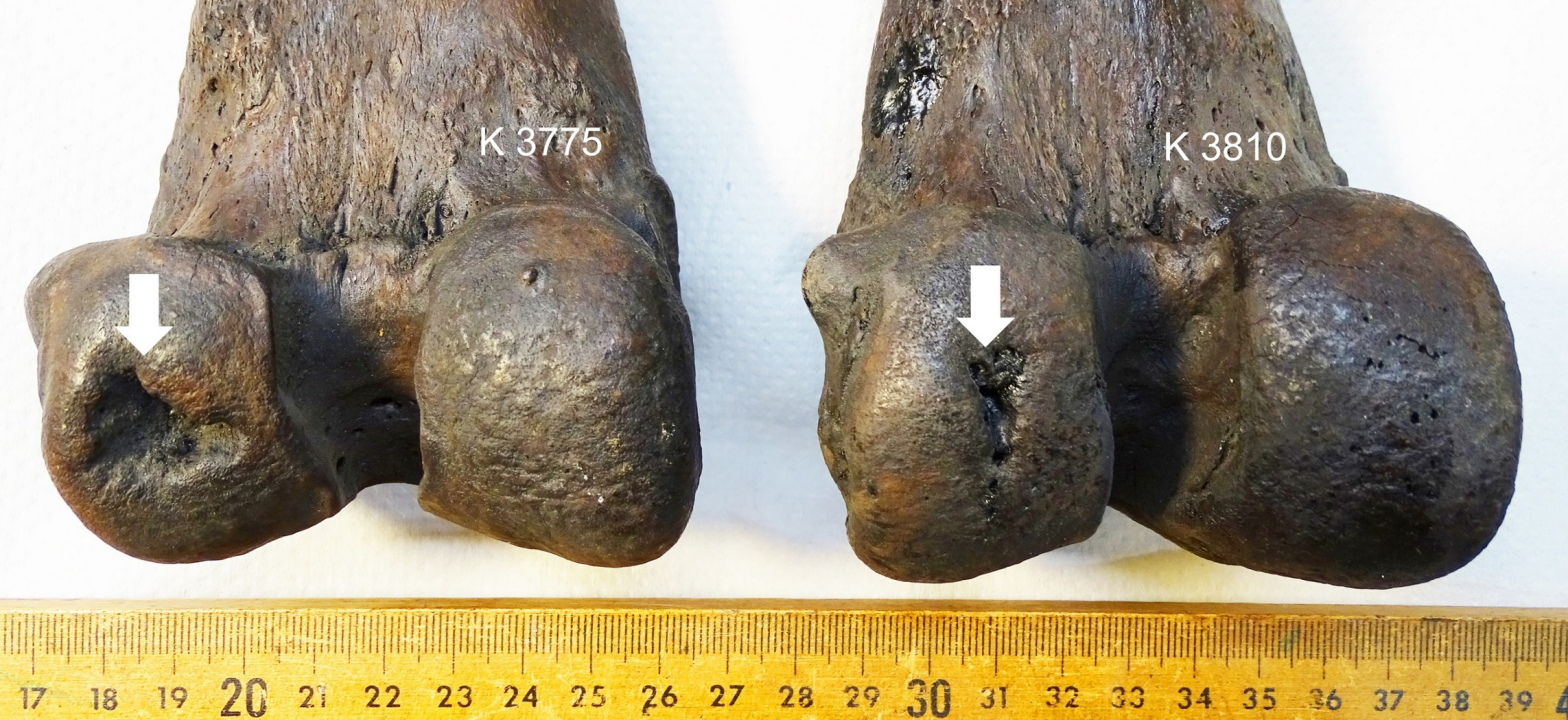
Large subchondral defects in adult *S*. *fatalis* specimen LACMHC K3775 and LACMHC K3810, no secondary osteoarthritis. The defect of LACM K 3775 is deeper and has sharper edges compared to LACM K 3810.

30 subchondral defects were documented in the adult femoral joint surfaces (7.3%), and 20 in the juvenile epiphyses (4.8%) (Figs 1 and 2). No subchondral defect resembling OC/OCD was found in the humeral and tibial joint surfaces.

30 defects were in the lateral condyle (14 in the center, 16 caudally), and 20 defects were in the medial condyle (18 in the center, 2 caudally) (Figs 1 and 2). In one case there were defects in the medial and lateral condyle, and one lateral condyle had a defect in the center and caudally. 50 cases in 834 femora give a prevalence of 6% subchondral defects in this collection of femora of *S*. *fatalis* found in Pits 3, 4, and 61/67. The prevalence across these three different deposits was similar, with 6.8% in Pit 3, 6% in Pit 4, and 5% in Pit 61/67.

**Fig 3:**
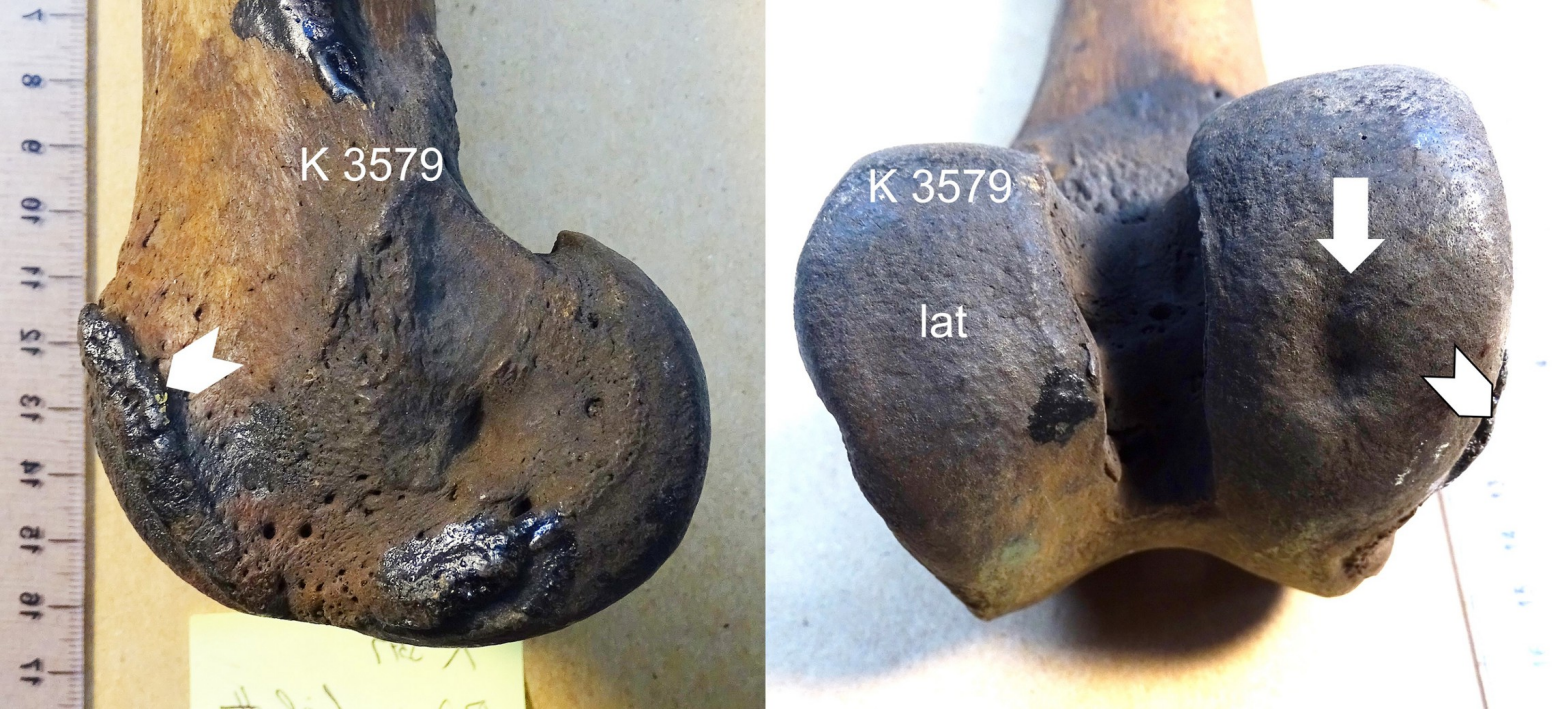
Medial center medium-sized defect (white arrow) and moderate osteophytes (arrow heads), *S*. *fatalis* LACMHC K3579.

**Fig 4:**
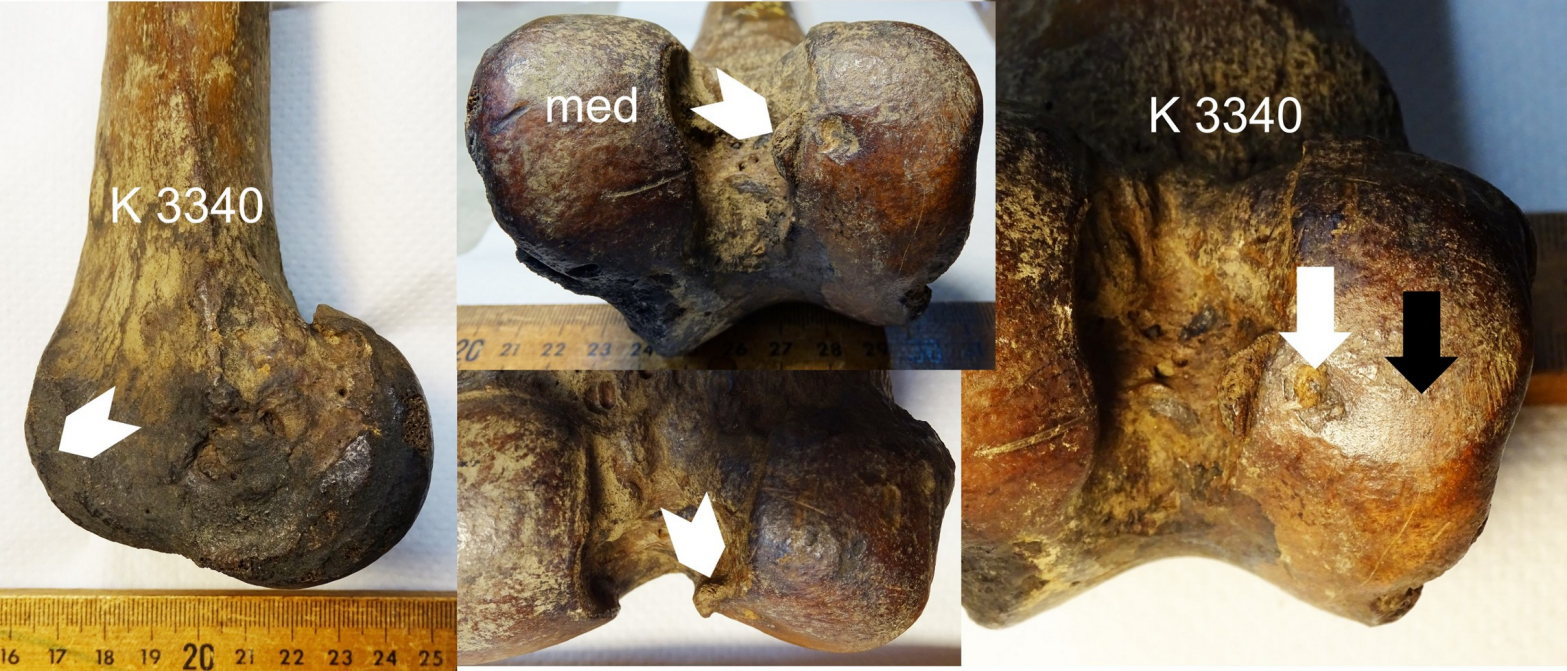
Lateral center small-sized defect (white arrow) and osteophytes (white arrow heads) indicating mild secondary osteoarthritis in this adult *S*. *fatalis* LACMHC K3340. The eburnation (black arrow) indicates a secondary damage to the opposite tibia joint surface and/or meniscus.

**Fig 5:**
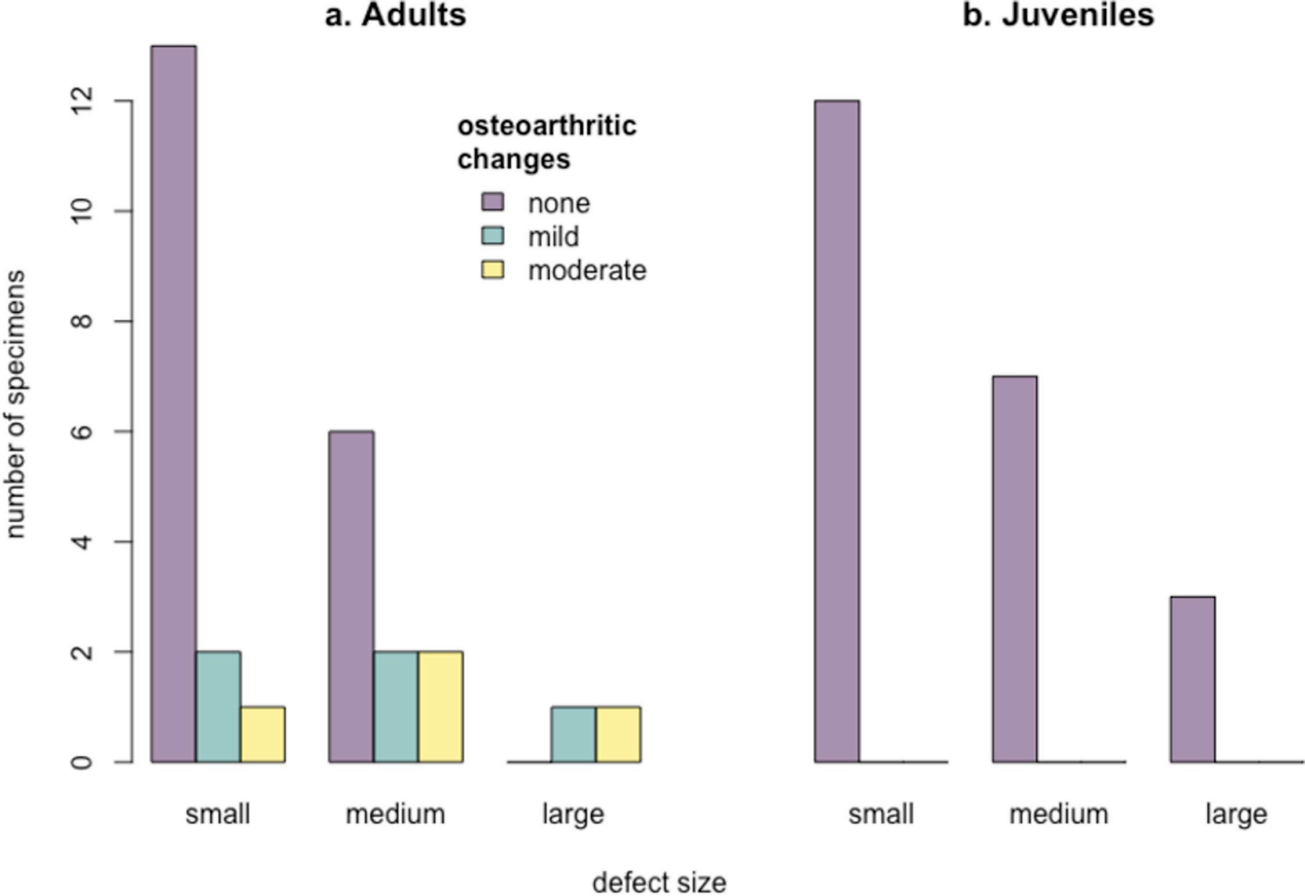
Barplots of defect size and osteoarthritic change in (a) adult and (b) juvenile *Smilodon fatalis* femoral joints only. (*S*. *fatalis* humeral joints showed no osteoarthritic change).

In 28 cases the defect size was small (56%), in 17 cases medium (34%), and in five cases large (10%). Further, in 9 adult stifle joints, osteoarthritis (OA) was documented: 5 cases with mild OA, and four cases with moderate OA (Figs 3 and 4). No case had developed severe OA. Five of the nine OA cases, including all moderate OA, had a defect in the medial center area. A weak though significant correlation, driven by most specimens with small defect size having no osteoarthritic change, was found between defect size and OA grading in the 28 adults (Fig 5A; Spearman’s rho = 0.399; *p* = 0.036). The correlation becomes weaker and statistically marginal when juveniles are included with adults in the analysis (Fig 5B; Spearman’s rho = 0.241; *p* = 0.091), as all 22 juveniles show no signs of OA (OA grading = none) regardless of defect size.

**Fig 6:**
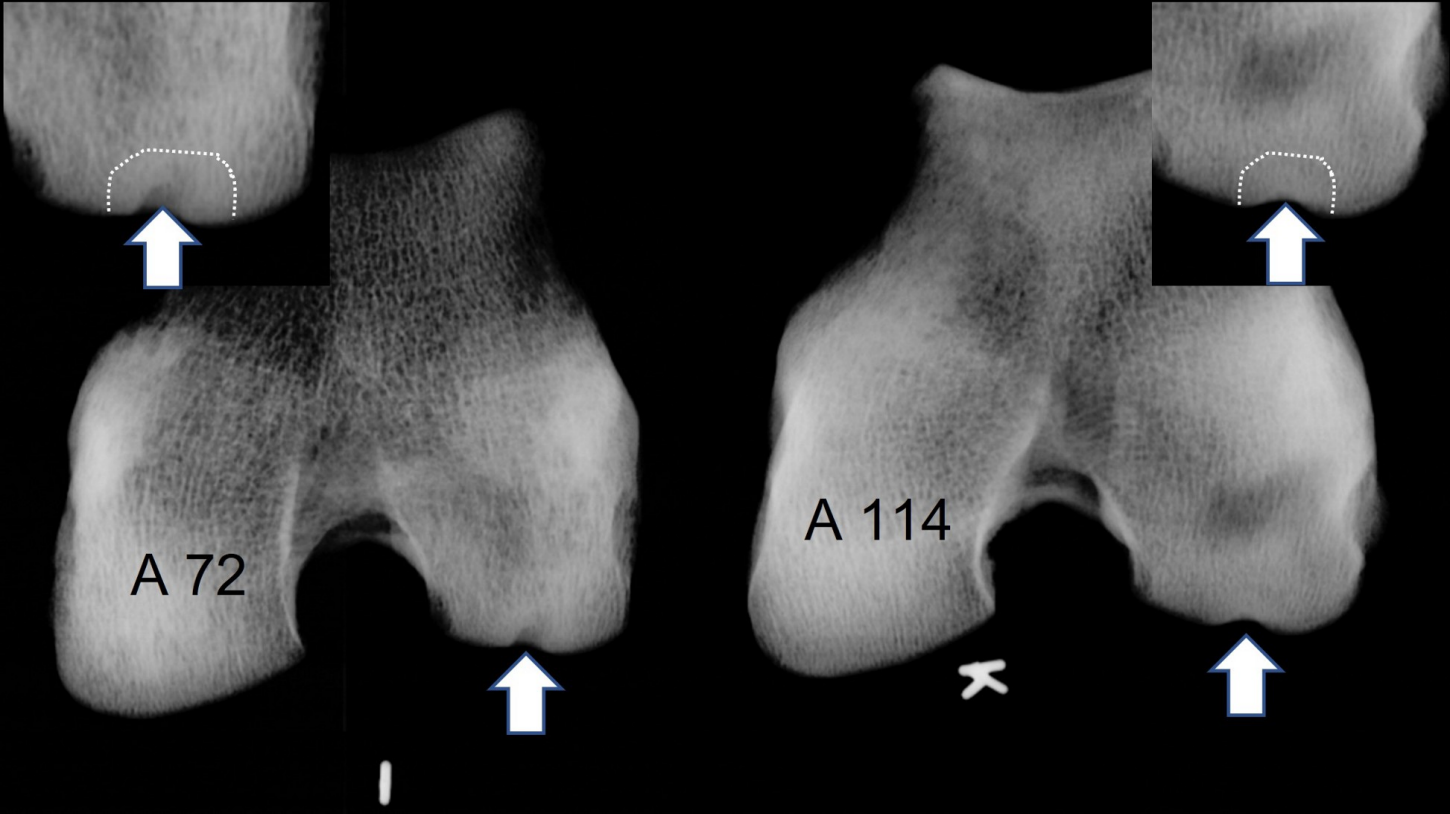
Radiographs of juvenile *S*. *fatalis* femoral joint surfaces LACMHC A72 and LACMHC A114 with a medium-sized lateral defect (white arrows) (same specimen as in Fig 1); the dotted line delineates the subchondral sclerosis surrounding the OCD defects.

The radiographs of two distal femoral epiphyses LACMHC A 72 and LACMHC A 114 show a subchondral bone defect in the condylar joint surface with a surrounding sclerosis looking very similar to OCD defects in modern domestic animals and the published cases of condyle OCD in *Panthera uncia* (Fig 6) [8, 9]).

### Aenocyon dirus

**Fig 7:**
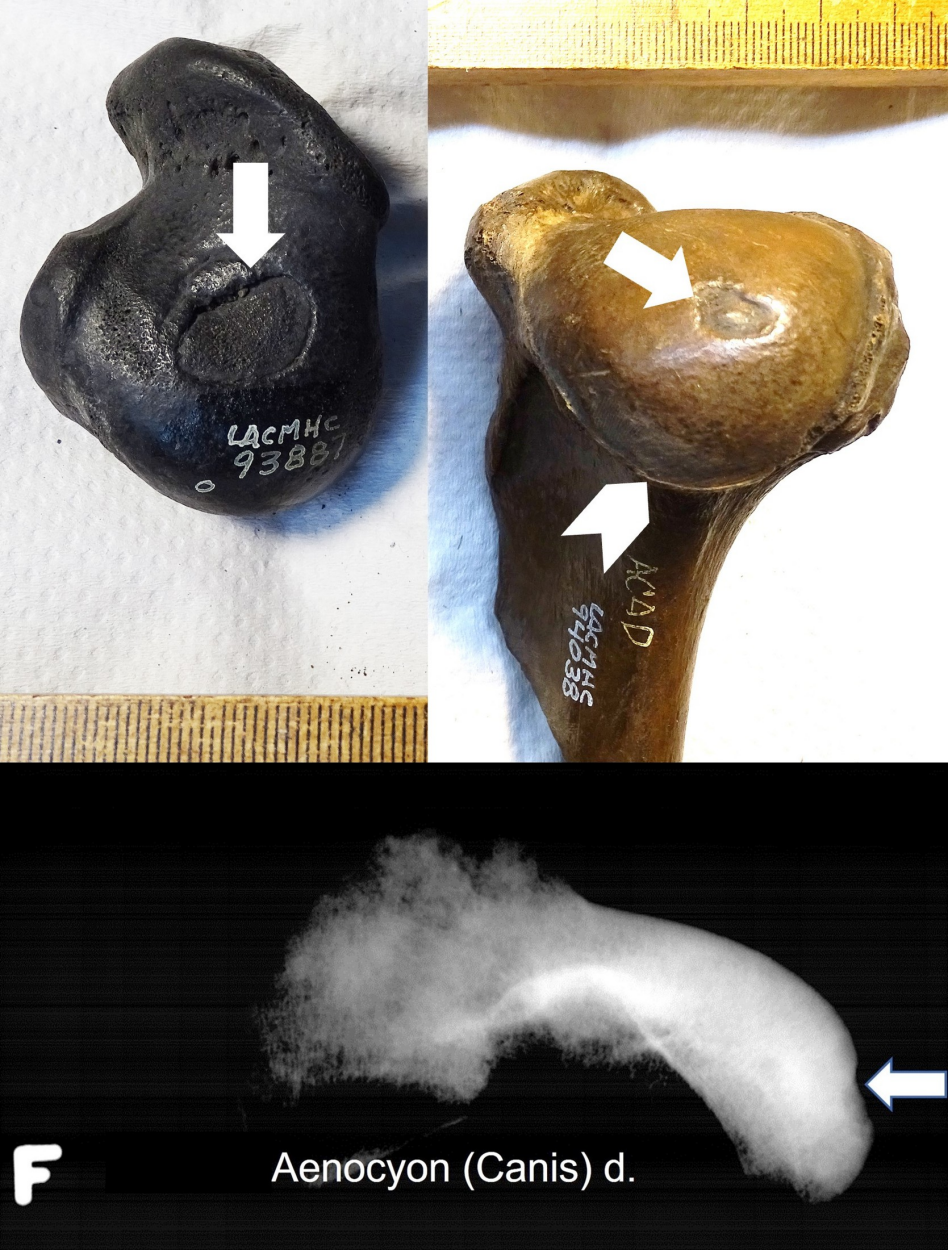
OC/OCD-like joint defects in the proximal humerus of *A*. *dirus* LACMHC 93887 and LACMHC 94038 (arrows) and the radiograph of *A*. *dirus* LACMHC 93743 humeral shoulder joint. The arrowhead indicates a ring of osteophytes indicating osteoarthritis.

116 adult proximal humeral joints and 126 juvenile proximal humeral epiphyses of *A*. *dirus* were inspected, and 11 subchondral defects found (Fig 7). Six defects were small, four medium-sized, and one defect was large. Three shoulders developed moderate OA. Eleven cases in 242 shoulders gives a prevalence of 4.5% subchondral defects.

**Fig 8:**
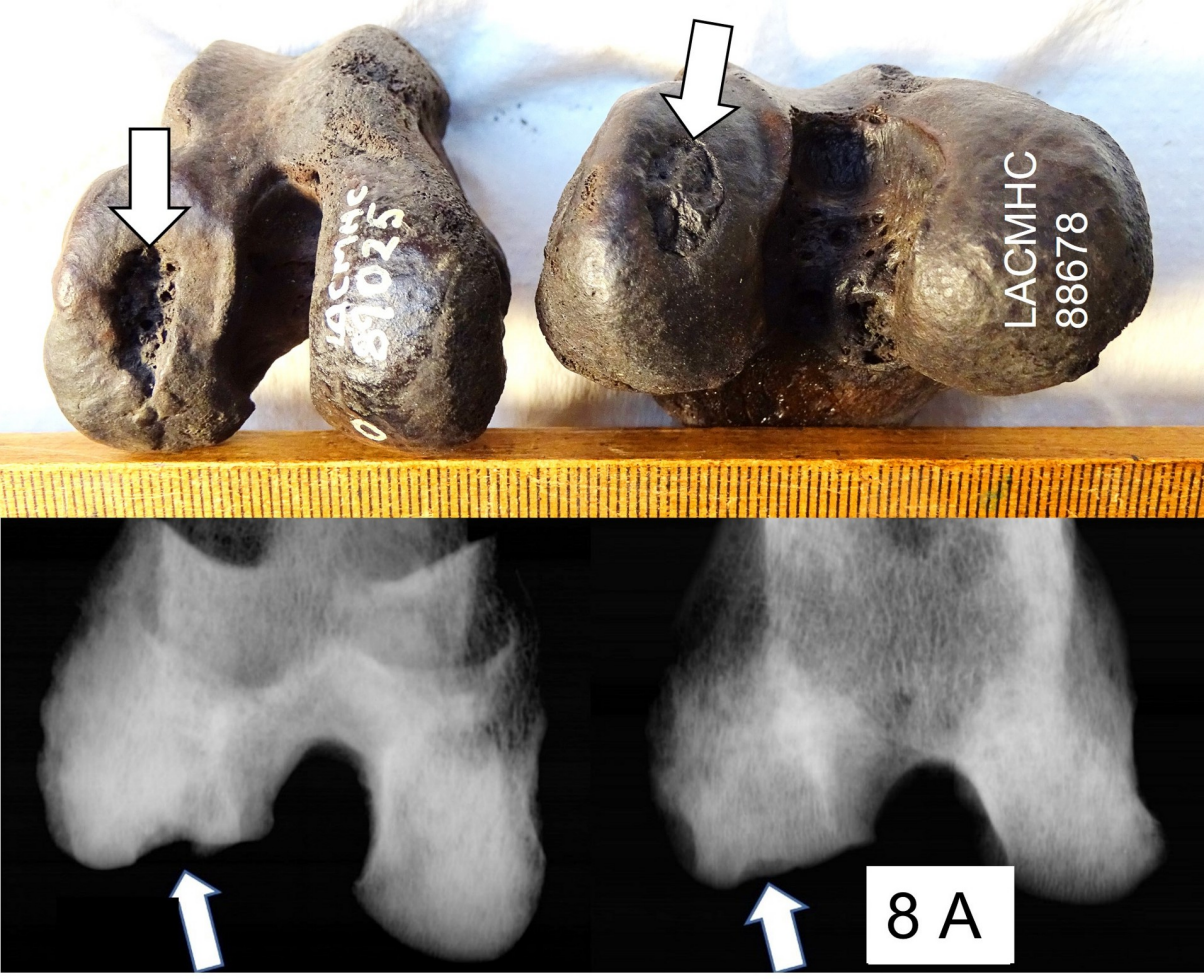
Large subchondral femoral defects in *A*. *dirus L*CMHC 89025 and LACMHC 88678, no secondary osteoarthritis. The defect of LACMHC 89025 is deeper and has sharper edges visible also on the corresponding radiographs.

266 femoral stifle joints of *A*. *dirus* and 170 proximal tibia joint surfaces were inspected. Seven subchondral defects were found in the femur (2.6%). No subchondral defect resembling OC/OCD was found in the tibial joint surfaces. Of the seven stifle defects, one was small, another was medium-sized, and five defects were large (Fig 8). Five defects were in the lateral condyle, two in the medial. Five stifles with defects developed mild OA; two were without OA. Radiographs of *A*. *dirus* LACMHC 89025 and 88678 stifle surfaces show defects very similar to the radiographic changes in modern dogs affected by stifle OCD (Fig 8).

**Fig 9:**
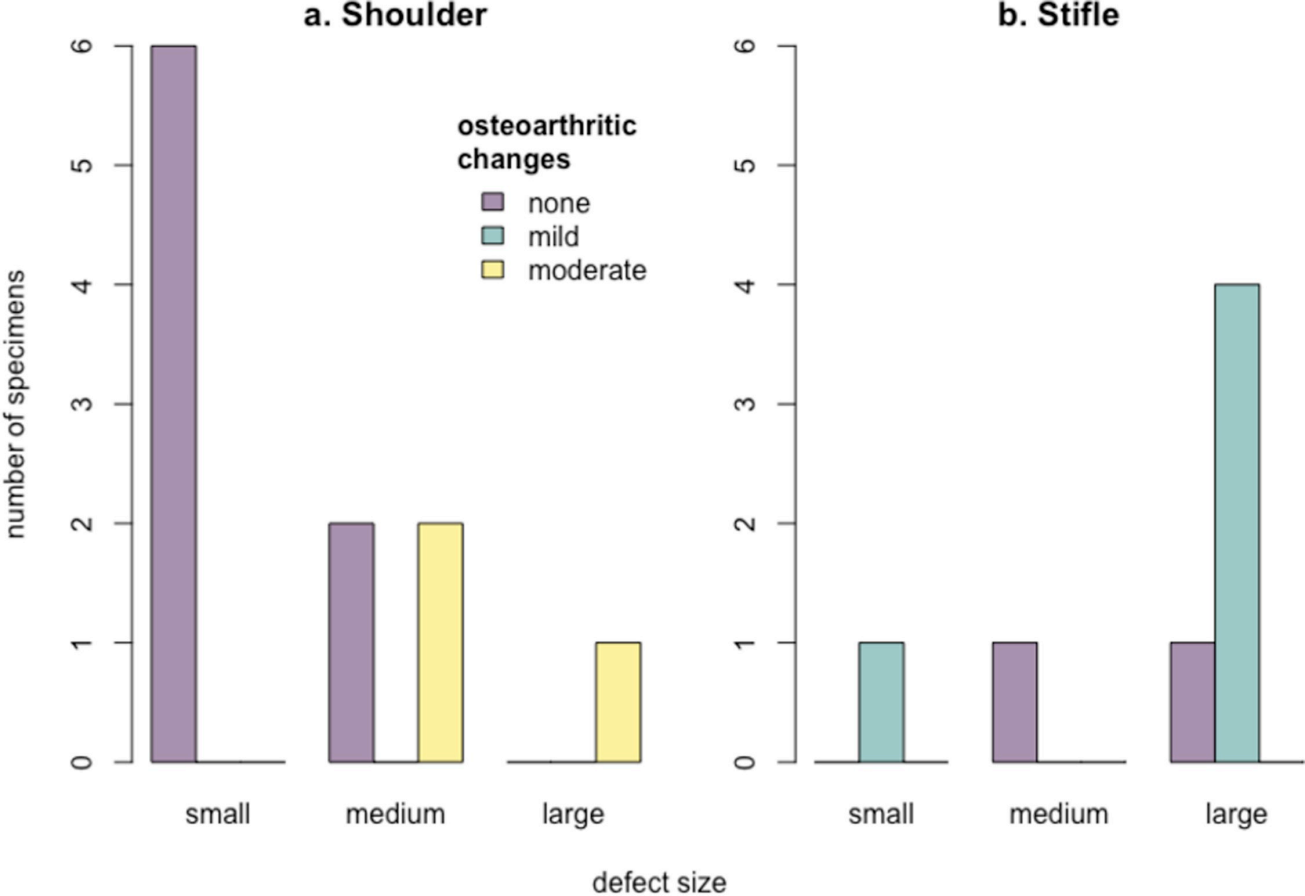
Barplots of defect size and osteoarthritic change in *Aenocyon dirus* (a) humeral joints (shoulder) and (b) femoral joints (stifle). Because of small sample size, both (a) and (b) show adults and juveniles together.

The *A*. *dirus* humeral joints showed a significant correlation between defect size and OA grading across both adults and juveniles (Fig 9A; Spearman’s rho = 0.724, *p* = 0.012), driven by all specimens with small defect size having no osteoarthritic change. As with *Smilodon*, all juveniles showed no osteoarthritic change regardless of defect size, though sample sizes were too small for adults (*n* = 6) and juveniles (*n* = 5) with subchondral defects to be analyzed separately. Meanwhile, the *A*. *dirus* femoral joints (*n* = 7; all adults save for one subadult) showed no relationship between defect size and degree of osteoarthritic change (Fig 9B; Spearman’s rho = 0.197, *p* = 0.672); most specimens showed a large defect size but mild osteoarthritic change.

## Discussion

Osteochondrosis (OC) is considered as a developmental disease, which can manifest as osteochondrosis dissecans (OCD) [2]. Involvement of diet, growth rate, certain hereditary factors, trauma, and vascular disorders makes OCD a multifactorial disease [4, 19].

Genetic predisposition is a risk factor in the development of OC including OCD [4, 20]. Several cases of OC have been reported to be genetically connected in humans [20, 21]. Two pairs of siblings were among five *Panthera uncia* treated for stifle OCD, possibly forming small family clusters of OCD as in humans [16, 17, 20, 21]. In dogs and horses, some breeds were observed to be more affected by OCD [22, 23]. Many reports have been published about the genetic background of OC/OCD, and the disorder is complex and controlled by several genes [21].

In modern dogs, OCD is mostly found in the shoulder joint, much less in the stifle [14]. However, our results indicate a more even OC/OCD pathology distribution between the shoulder and the stifle in *A*. *dirus* (Fig 9). In humans and the rare cases of domestic cats and the snow leopard (*P*. *uncia*), the femoral condyles were commonly affected [15–17, 21, 24]; the same seems to be true for the saber-toothed cat. Meanwhile, in previously documented cases of modern felids with OC/OCD, only the lateral condyle was affected [15–17]. In contrast, both femoral condyles of the Rancho La Brea *S*. *fatalis* had lesions looking like OC/OCD like in modern dogs, both in the center of the condyle and more caudally. Medial center was the single most affected condyle area followed by lateral caudal, making *S*. *fatalis* different in OC/OCD pattern from the felid species living today. No subchondral defects looking like OC/OCD were found in the tibial surfaces, as it is the same for modern dogs and cats [14].

The prevalence of 6% affected femora in the growing and adult *Smilodon fatalis* population is high, compared to humans with a prevalence of 15–29 per 100,000 (0.015–0.029%) and a prevalence of 0.05% of stifle OCD cases in domestic dogs [14, 21, 24]. At 2.6% in the femur, the detected stifle OCD prevalence in *A*. *dirus* is lower than that in *S*. *fatalis*. But, with 4.5% in the proximal humerus, prevalence in *A*. *dirus* is similarly high as in modern affected dog breeds such as the Border Collie and the Greater Swiss Mountain Dog [25]. The development of osteoarthritis (OA) in the OCD-affected dog stifle and shoulder is normally slow and moderate at a young age, and the OA found in the *A*. *dirus* shoulders and stifles does not differ much from the modern dog [14].

Only nine of 30 subchondral defects in adult *S*. *fatalis* showed mild to moderate osteoarthritic pathologies (Fig 5). A possible explanation for the low incidence of severe osteoarthritis found in the stifles with a defect is the clinical experience that, in humans and modern dogs, early stages of OC (OC *latens*) can heal in patients before it develops into severe OCD and clinical osteoarthritis [24, 26]. When OC development induces cartilage lesions, the cartilage progenitor cells within the articular tissues will initiate the formation of cells that will regenerate and repair the damaged tissues [27]. Another explanation could be the fact that the victims of the RLB asphalt seeps did not reach their natural life expectancy, their lives cut short by asphaltic entrapment: dental examination shows that most fossilized carnivores at RLB were young adults [28]. It remains unknown how OCD would have affected the joints in older animals. We can assume that more joints would have developed advanced osteoarthritis during a longer life span, especially the joints with large and deep subchondral defects in the weight-bearing cartilage surface.

Evidence of OCD in these predators suggests that the disease did not hamper their ability to survive, even though survival to adulthood depends on access to large prey that would be difficult to hunt with impaired locomotion. Compared to the OCD defect size in the clinically symptomatic snow leopards [16, 17], the defects in *S*. *fatalis* were mostly small, with only 10% being larger than 12mm, which would have increased the chance for spontaneous healing without developing an OCD flap. As well, social behavior may have played a role: fossils from the Quaternary deposits of the Santa Elena Peninsula in Ecuador indicate that subadult *Smilodon* stayed with their mother for a long time, as is common in large Felidae today [29]. Even if the defects in the femoral cartilage surface would have clinically affected the subadult saber-toothed cat as in the reported subadult snow leopards, survival would have been supported by the mother’s hunting and protection. Similarly, the dire wolf’s pack behavior—inferred to be like that in living grey wolves, *Canis lupus*—probably also supported wounded or sick members of their group [30].

**Fig 10:**
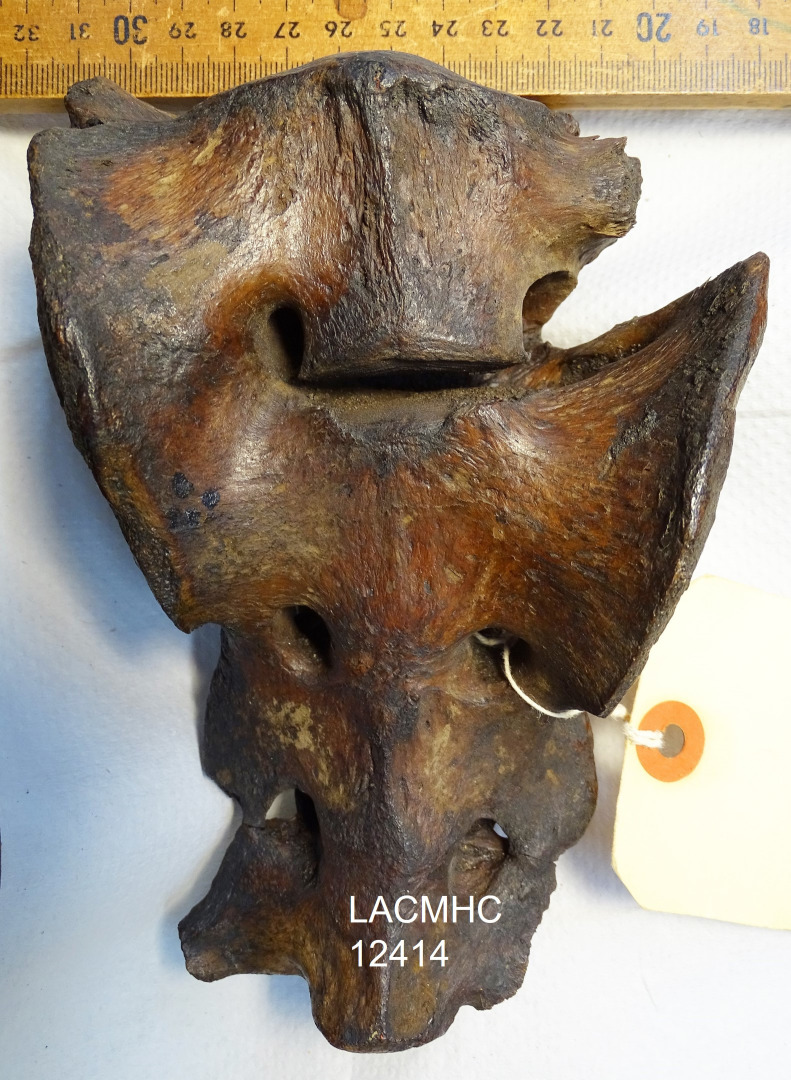
Transitional lumbosacral vertebra of adult *S*. *fatalis* LACMHC 12414.

As osteochondrosis is a developmental disease with an underlying genetic basis, its prevalence may suggest a genetic response to environmental stress, inbreeding, or other large-scale factors. Thus far, no ancient DNA has been recovered from Rancho La Brea [31], so it is not possible yet to estimate the degree of interrelatedness of the *S*. *fatalis* and *A*. *dirus* populations that lived in the Pleistocene of Los Angeles. However, during the period preserved at Rancho La Brea—just preceding the megafaunal extinctions around 12,000–10,000 years ago—*S*. *fatalis* and *A*. *dirus* were approaching the end of their species’ lifespans. Today, endangered species experience reduced population densities and associated complications from inbreeding. For example, the Florida panther (*Puma concolor coryi*) demonstrates a high frequency of kinked tails and joint disease, morphological anomalies accompanying a suite of inbreeding markers—including congenital heart defects, cryptorchidism, and vulnerability to pathogens—that indicate a highly stressed population at risk [32]. A high degree of inbreeding can also increase the frequency of spinal malformation, like transitional lumbo-sacral vertebra (LSTV) [33, 34]—examples of which, in addition to the femoral defects resembling OC/OCD, are found in the RLB collection of *A*. *dirus* and *S*. *fatalis* bones (e.g., Fig 10). Based on conservation genetics research on threatened extant species [e.g., 35, 36], it is possible that large-scale ecological and/or environmental change toward the end of the last Ice Age caused groups of large herbivores and carnivores to become more and more geographically isolated, altering demography and accelerating inbreeding prior to their extinction [37]. This inbreeding process increases the genetic load of the population, manifesting in the increased occurrence of inherited diseases. Recent studies using DNA analysis to calculate the inbreeding factor F_adj_ in modern domestic dogs showed an overall very high degree of inbreeding, and OCD and LSTV are a common disease in many dog breeds [38].

The current study is limited by the fact that only disarticulated and unassociated bones with the joint surfaces could be examined, not the joint function and the cartilage or the surrounding soft tissues. We can only compare the subchondral joint surface defects in the extinct *S*. *fatalis* and *A*. *dirus* with similar joint surface defects found in living animals today. Despite this limitation, our results do not support our prediction that OC/OCD was a rare pathology in the Pleistocene *S*. *fatalis* and *A*. *dirus*. We found a high prevalence of subchondral defects in the femoral and humeral joint surfaces resembling OC/OCD found in humans and other mammals. Research is needed to determine if these defects also affected *S*. *fatalis* and *A*. *dirus* in other areas of North and South America, illuminating potential pathological patterns as these two species approached extinction.

The genetic background of osteochondrosis seems to have existed long before domestication of animals by humans, affecting vertebrates with a cartilage cap over their articular surfaces ranging from hadrosaurs in the Late Cretaceous to *Smilodon fatalis* and *Aenocyon dirus* in the Pleistocene [39]. In modern times, OCD affects birds and different mammals including humans, if the conditions—for example, degree of inbreeding, fast growth, or high level of sporting activity in subadults—allow the disease to be expressed, causing painful joint disease as consequence.

## Supporting information

S1 Table Distal femoral joint surface with a subchondral defect in *Smilodon fatalis* and *Aenocyon dirus*. (PDF)

S2 Table Proximal humeral joint surface with a subchondral defect in *Aenocyon dirus*. (PDF)

S3 Table Stifle joint (tibia and femur) with non-OCD related pathologies in *Aenocyon dirus*. (PDF)

S4 Table Stifle joint (tibia and femur) with non-OCD related pathologies in *Smilodon fatalis*. (PDF)
