# Supplementary material for: Subchondral defects resembling osteochondrosis dissecans in joint surfaces of the extinct saber-toothed cat Smilodon fatalis and dire wolf Aenocyon dirus
Source: PLoS One. 2023 Jul 12;18(7):e0287656. doi: 10.1371/journal.pone.0287656 (PMC10337945; doi:10.1371/journal.pone.0287656)
Supplement: S1 Table — (PDF) [file pone.0287656.s001.pdf]

S1 Table: Distal femoral joint surface with a subchondral defect in *Smilodon fatalis* and *Aenocyon dirus.*

Specimen number	Adult A or Juvenile J	OCD size	Localisation	OA grading
Smilodon fatalis				
LACMHC K3748	A	2	medial caudal	none
LACMHC K3722	A	1	medial caudal	none
LACMHC K3718	A	1	lateral caudal	none
LACMHC K3762	A	2	lateral caudal	none
LACMHC K3329	A	1	lateral caudal	none
LACMHC K3434	A	2	lateral caudal	none
LACMHC K3446	A	1	lateral caudal	none
LACMHC K3395	A	1	lateral caudal	none
LACMHC K3404	A	1	lateral	none
LACMHC A456	A	1	medial	none
LACMHC K3373	A	1	medial	none
LACMHC K3303	A	1	medial	none
LACMHC K3272	A	1	lateral	none
LACMHC K3288	A	2	medial	none
LACMHC 43132	A	1	lateral	none
LACMHC K3585	A	2	medial	none
LACMHC A20	A	1	medial	none
LACMHC K3218	A	1	medial	none
LACMHC K3235	A	2	medial	none
LACMHC 111246	A	1	medial	mild
LACMHC A455	A	2	lateral caudal	mild
LACMHC K3389	A	2	medial caudal	mild
LACMHC K3572	A	1	lateral caudal	mild
LACMHC K3234	A	3	medial	moderate
LACMHC 8946	A	1	medial	moderate
LACMHC 9116	A	2	medial	moderate

LACMRLP R15239	A	3	lateral	mild
LACMHC K3579	A	2	medial	moderate
LACMHC K3810	Subadult	3	medial	none
LACMHC K3195	Subadult	1	lateral caudal	none
LACMHC A72	J	1	lateral caudal	none
LACMHC A114	J	1	lateral caudal	none
LACMHC A375	J	2	medial	none
LACMHC A219	J	1	lateral caudal	none
LACMHC A271	J	1	lateral caudal	none
LACMHC A317	J	2	lateral caudal	none
LACMHC A236	J	1	medial	none
LACMHC A136	J	1	lateral caudal	none
LACMHC A67	J	1	lateral	none
LACMHC A280	J	1	medial	none
LACMHC A242	J	2	lateral	none
LACMHC 43183	J	1	lateral	none
LACMHC A257	J	2	lateral	none
LACMHC A135	J	1	lateral	none
LACMHC A345	J	2	lateral	none
LACMHC A251	J	3	lateral and medial	none
LACMHC A208	J	2	lateral caudal	none
LACMHC 43310	J	1	lateral caudal	none
LAMCHC 53136	J	3	lateral caudal	none
LACMRLP R41717	J	2	lateral	none

Aenocyon dirus

LACMHC H504	A	2	medial	none
LACMHC H246	A	3	lateral	none
LACMHC 88819	A	3	medial	mild

LACMHC 88678	A	3	lateral	mild
LACMHC 88262	A	1	lateral	mild
LACMHC 88213	A	3	lateral	mild
LACMHC 89025	Subadult	3	lateral	mild
