# Supplementary material for: Subchondral defects resembling osteochondrosis dissecans in joint surfaces of the extinct saber-toothed cat Smilodon fatalis and dire wolf Aenocyon dirus
Source: PLoS One. 2023 Jul 12;18(7):e0287656. doi: 10.1371/journal.pone.0287656 (PMC10337945; doi:10.1371/journal.pone.0287656)
Supplement: S2 Table — (PDF) [file pone.0287656.s002.pdf]

S2 Table: Proximal humeral joint surface with a subchondral defect in *Aenocyon dirus.*

Specimen number	Ault A or Juvenile J	OCD size	OA grading
LACMHC I6185	A	1	none
LACMHC 93982	A	2	moderate
LACMHC I5964	A	1	none
LACMHC I5832	A	2	none
LACMHC 94038	A	2	moderate
LACMHC 94166	A	3	moderate
LACMHC 93887	J	1	none
LACMHC 93743	J	1	none
LACMHC 93764	J	1	none
LACMHC 93801	J	2	none
LACMHC 93802	J	1	none
