# Supplementary material for: Subchondral defects resembling osteochondrosis dissecans in joint surfaces of the extinct saber-toothed cat Smilodon fatalis and dire wolf Aenocyon dirus
Source: PLoS One. 2023 Jul 12;18(7):e0287656. doi: 10.1371/journal.pone.0287656 (PMC10337945; doi:10.1371/journal.pone.0287656)
Supplement: S3 Table — (PDF) [file pone.0287656.s003.pdf]

S3 Table: Stifle joint (tibia and femur) with non-OCD related pathologies in *Aenocyon dirus.*

Specimen Number	OA grade	Comment
**Tibia**		
LACMHC H1356	mild	
LACMHC H1417	mild	
LACMHC 7371	moderate	osteophyte insertion CrCL
LACMHC H2191	moderate	medial buttress
LACMHC H2046	moderate	medial buttress
LACMHC 7312	moderate	medio-proximal crest trauma
LACMHC H1665	moderate	lateral plateau eburnation
LACMHC 7394	moderate	lateral plateau eburnation
LACMHC H1511	moderate	
LACMHC 7351	moderate	
LACMHC 7369	severe	medial buttress
LACMHC 7330	severe	medial plateau eburnation
LACMHC 69201	severe	lateral plateau eburnation
LACMHC 7343	severe	medial buttress, caudal osteophyte medial
**Femur**		
LACMHC 7466	mild	
LACMHC 7401	mild	
LACMHC 7487	mild	
LACMHC 7500	mild	
LACMHC H755	mild	OA mainly in the notch
LACMHC H866	mild	
LACMHC H569	mild	OA mainly in the notch

LACMHC H594	mild	OA mainly in the notch
LACMHC H312	moderate	
LACMHC12826	severe	condylar eburnation
LACMHC 7422	severe	
LACMHC 7516	severe	
LACMHC12931	severe	extreme changes
LACMHC 7461	severe	condylar eburnation
LACMHC13384	severe	patella problem
