# Supplementary material for: Subchondral defects resembling osteochondrosis dissecans in joint surfaces of the extinct saber-toothed cat Smilodon fatalis and dire wolf Aenocyon dirus
Source: PLoS One. 2023 Jul 12;18(7):e0287656. doi: 10.1371/journal.pone.0287656 (PMC10337945; doi:10.1371/journal.pone.0287656)
Supplement: S4 Table — (PDF) [file pone.0287656.s004.pdf]

S4 Table: Stifle joint (tibia and femur) with non-OCD related pathologies in *Smilodon fatalis.*

Specimen number	OA grade	Comment
**Tibia**		
LACMHC K4460	mild	
LACMHC K4194	mild	
LACMHC K4096	mild	
LACMHC 43802	mild	
LACMHC K4132	mild	
LACMHC K4319	mild	
LACMHC K4196	mild	
LACMHC K4577	mild	
LACMHC K4473	mild	
LACMHC K4650	mild	
LACMHC K4305	severe	
LACMHC K3899	severe	
**Femur**		
LACMHC 8943	mild	notch stenosis
LACMHC K3807	mild	
LACMHC K3161	mild	
LACMHC K3514	mild	
LACMHC K3361	mild	OA in the notch
LACMHC K3664	mild	OA laterally
LACMHC111246	mild	
LACMHC K3686	moderate	lateral condyle eburnation rim +
LACMHC K3340	moderate	lateral condyle eburnation rim ++
LACMHC K6947	severe	lateral condyle eburnation ++
